# Stakeholders’ perspectives on online interventions to improve mental health in eating disorder patients and carers in Germany

**DOI:** 10.1093/eurpub/ckab057

**Published:** 2021-07-07

**Authors:** Juliane Schmidt-Hantke, Bianka Vollert, Franziska Hagner, Ina Beintner, Kristian Hütter, Martina Nitsch, Corinna Jacobi, Karin Waldherr

**Affiliations:** 1 TU Dresden, Faculty of Psychology, Institute of Clinical Psychology and Psychotherapy, Dresden, Germany; 2 Ferdinand Porsche FernFH-Distance Learning University of Applied Sciences, Wiener Neustadt, Austria

## Abstract

**Background:**

Eating disorders are causing severe consequences for those affected as well as a high burden for their carers. Although there is a substantial need for psychological assistance, different factors are hindering access to support. Internet-based interventions can help to overcome these barriers. To date, there is only little knowledge on attitudes of potential users, facilitators (e.g. psychologists) and decision makers (e.g. health insurances) regarding these interventions.

**Methods:**

We conducted focus groups with potential users (*N* = 30) and semi-structured interviews with potential decision makers (*N* = 4). Potential facilitators (*N* = 41) participated in an online survey. Stakeholders’ experiences, attitudes, and their needs regarding Internet-based interventions for eating disorder patients and carers were assessed. Furthermore, hindering and fostering factors related to reach, adoption, implementation and maintenance were analyzed.

**Results:**

About two-thirds of the participating facilitators have heard or read about Internet-based interventions in general. In contrast, the other stakeholders mentioned to have no or little experience with such interventions. Factors like anonymity, availability and cost-effectiveness were seen as major advantages. Also disadvantages, e.g. lack of personal contact, limitations by disease severity and concerns on data safety, were mentioned. Stakeholders stated the need for interventions which are usable, evidence-based, tailored and provide personal support.

**Conclusion:**

Stakeholders considered Internet-based programmes to have more advantages than disadvantages. Effort should be put in providing systematic education to address prejudices. When offering an online intervention, stakeholders’ needs, as well as a continuous evaluation and adaptation, have to be taken into account.

## Introduction

Eating disorders are serious conditions with both physical and psychological problems resulting in an impaired quality of life in those affected.^1^ Thus, treatment is indicated as early as possible. One major barrier to treatment is the limited treatment capacity of (mental) health care providers. Even in countries with good health care coverage like in Germany, immediate or prompt access to mental health services cannot be taken for granted. A recent report from the ‘Bundespsychotherapeutenkammer’ (German Psychotherapists’ Association) indicated that people with mental health disorders have to wait almost 20 weeks to start face-to-face cognitive-behavioural therapy.^2^ A further, currently relevant factor to be considered is the limited availability of face-to-face treatments due to the COVID-19 pandemic. 

Besides the availability and accessibility of treatment, factors like shame and stigma, low treatment motivation, denial of the disorder or lack of knowledge hinder help-seeking behaviours of those affected.^3^ Accordingly, the number of eating disorder patients undergoing treatment is low, especially for bulimic disorders.^4^ On the other hand, patients who achieve an early reduction of symptoms are more likely to also achieve remission,^5^^,^^6^ and rapid response to treatment is associated with better outcomes.^7^ Not receiving adequate treatment (or any kind of treatment at all) can cause chronification and far-reaching consequences for both the patient and the health care system.

Considering the social consequences of an eating disorder, it is important to highlight that eating disorders do also affect the quality of life of the whole family. Carers of sufferers from an eating disorder experience intense distress and burden^8^ and show clinically significantly elevated levels of depression and anxiety.^9^ As carers play an important role in recovery, a lack of information and skills can cause problematic carer behaviours, which may worsen or maintain the disease. Although they are often highly motivated to support their loved ones,^10^ carers experience their needs as unconsidered by health care professionals and self-help organizations, e.g. lack of information about the eating disorder and help in dealing with the disease.^11^ Furthermore, there is a lack of support, especially in remote and rural areas.

To overcome barriers like limited access to mental health services, fear of stigmatization as well as a long waiting time, providing easily accessible help is needed. Evidence-based Internet-delivered approaches can facilitate access to mental health care as they are accessible anytime and anywhere and can be seen as a low-threshold service. Further advantages include cost-effectiveness compared with face-to-face treatment,^12^ anonymity and a greater user control.^13^ Besides this, Internet-based interventions are often the only way to provide mental health care during times of a pandemic leading to physical distancing.

Previous studies found Internet-based interventions to be effective in improving (general) eating disorder psychopathology/symptoms and quality of life^14–21^ and in decreasing risk factors.^17^^,^^19^ In this context, these interventions were superior to waiting lists.^22^ Dölemeyer *et al.*^13^ stated CBT-based online treatments targeting eating disorders to be a good alternative for face-to-face treatment. Internet-based interventions targeting carers of patients with an ED are rare. Existing interventions have been shown to improve negative health impacts of caregiving in carers, such as negative experiences of caregiving, as well as to reduce carers’ anxiety and depression. They can also help carers to meet their own needs.^23^^,^^24^

Despite showing the effectiveness of Internet-based interventions for eating disorders it is also necessary to concern acceptability of and expectations towards these interventions. Grover *et al.*^24^ reported that participants of a web-based intervention for carers of people suffering from anorexia nervosa [overcoming anorexia online (OAO)]^25^ rated this intervention as acceptable and useful. Participants perceived it as an advantage to be flexible in terms of location and time and independent from making an appointment. With regard to eating disorder patients, guided CBT-based online treatments were found to be acceptable to adult female patients suffering from a binge eating disorder.^26^ Basterfield *et al.*^27^ also examined the view of eating disorder patients on the use of Internet-based technology. First, patients stated concerns regarding safety, e.g. how to find credible and reliable material. They stated resources as helpful when they were recommended by clinicians or guided by professionals. The second identified theme was connection with a support system, e.g. via messaging with a professional. Concerning this, participants noted that face-to-face interaction can lead to social anxiety. The third identified aspect was technology development. Patients suggested which tools would be helpful in the future, e.g. meal support or tracking of recovery activities. They also mentioned factors like customizability or convenience.

Besides considering perspectives of potential users of Internet-based interventions, also those of potential providers have to be taken into account, as their attitudes have a considerable impact on the efficacy of an intervention. Since no previous study assessed attitudes of professionals towards Internet-based interventions for eating disorders, we will report their attitudes towards online interventions in general. Wells *et al.*^28^ examined concerns and considerations of professionals like psychologists regarding online mental health treatment in general. 82% of the participants expressed concerns; the primary concern was on confidentiality of client information. They also reported concerns about liability, misinformation provided by patients and a lack of qualification for conducting online treatment. Some professionals underlined that face-to-face contact is necessary for an effective psychotherapy. Clinicians also expressed fear of losing their work if Internet-based treatment is able to replace face-to-face treatment.^12^ Beside these concerns, professionals also stated advantages of online support, e.g. to reach rural populations or people with a limited mobility due to an illness.^28^ Klasen *et al.*^29^ reported that despite concerns due to the lack of face-to-face contact in Internet-based interventions, a stable therapeutic relationship can be built. To tackle the reported prejudices against e-health interventions, educational work has proved to be effective.^30^ Van der Vaart *et al.*^31^ were examining attitudes of therapists and patients regarding online and blended therapy for depression. Although they expressed the need to see their patient/therapist face-to-face as well as worries about misinterpretation due to the lack of non-verbal communication, both participant groups assumed that online and blended therapy will have more benefits than disadvantages. However, referring to a survey conducted in Germany, a lot of people of the general population considered the Internet as an important source of health information, but only a few are aware of the existence of Internet-based psychological counselling and interventions. About half of the participants were willing to make use of online-counselling and less than 10% were willing to use online-based treatments. There was a clear preference to use face-to-face treatments.^32^ It is assumed that this preference has changed in the past months due to the COVID-19 pandemic and the resulting need for online mental health care. A recent study examines psychotherapists attitudes towards and experiences with Internet-based psychotherapy in times of the current pandemic. Many psychotherapists were forced to offer psychotherapy online in these times. Despite various obstacles (e.g. technical problems, risk of getting distracted and difficulties to read patients emotions), psychotherapists reported reasonably positive attitudes towards Internet-based psychotherapy. These attitudes were influenced by different factors, such as therapy modality, clinical experience, online psychotherapy experience, perceived patient experiences or experiences during the COVID-19 pandemic. Asking patients, mainly positive (62.8%) experiences with Internet-based psychotherapy were reported. Only 7.6% of the patients stated negative experiences.^33^

On the one hand, it seems to be essential to include attitudes and perspectives of potential users in the development of Internet-based interventions to meet their needs and to mitigate intervention drop-out rates. As reported by Dölemeyer *et al.*^13^ treatment drop-out rates in Internet-based interventions for eating disorders were partially high and ranging from 9% to 47.2%. Grover *et al.*^24^ reported that 72.7% of participants allocated to the abovementioned Internet-based intervention OAO^25^ filled the post-assessment. Only 60.6% filled the follow-up questionnaires. On the other hand, it is also important to involve decision-makers and facilitators to increase reach and adoption and to improve general conditions for the provision of e-health interventions.

To the best of our knowledge, to date, no study was conducted that focused on general attitudes and perspectives of carers from individuals suffering from an eating disorder towards Internet-based self-help interventions. Most findings about acceptability of these interventions are based on data of participants completing an intervention and therefore possibly positively biased as unsatisfied participants are more likely to become a non-completer.^34^ Additionally, there were no previous findings on the view of decision-makers although they are very important when it comes to sustain a programme. The aim of the current stakeholder survey was therefore to include representatives of target groups, potential decision makers and facilitators with the aim to give an overview on their previous experiences and their current attitudes towards Internet-based interventions for eating disorder patients as well as carers of people suffering from an eating disorder. Furthermore, hindering and fostering factors for reach, adoption, implementation and maintenance with regard to these Internet-based interventions will be presented. The stakeholder survey was carried out in the context of the ICare project, financed by the EU (GA No. 634757). The aim of ICare was to implement various Internet-based interventions for the prevention and treatment of mental health problems in six European countries.^35^

## Methods

### Study design

The overall ICare stakeholder survey focused on Internet-based universal and targeted prevention of common mental health problems.^36^ For the current study which aimed to examine perspectives on online self-help interventions for people suffering from an eating disorder and their carers, some study-specific adaptations were necessary. In correspondence with the overall stakeholder survey, three stakeholder groups were approached, namely (i) potential users (eating disorder patients and their carers), (ii) potential facilitators (individuals who work with potential users and might support the implementation of the programmes) and (iii) decision-makers (stakeholders at the governing level). Since each of the stakeholder groups has its specific characteristics, we applied a concurrent mixed-methods design in accordance with the overall stakeholder survey. Therefore, interviews with decision-makers, focus groups with potential users as well as an online survey with potential facilitators were conducted. The identification of stakeholder groups and the concurrent mixed-methods design is described in more detail in the design paper of the overall stakeholder survey in this supplement.^36^

### Instruments

The questionnaire items as well as the topic guides used in the telephone interviews and focus groups were aligned as closely as possible to those of the overall ICare stakeholder survey^36^ and were partly based on the RE-AIM dimensions reach, adoption, implementation and maintenance.^37^ They consisted of questions addressing the following issues: attitudes towards Internet-based interventions in general as well as in particular for eating disorder patients and their carers, previous experiences with and needs towards Internet-based interventions. In addition, hindering and fostering factors with regards to the above mentioned RE-AIM measures were investigated. To assess the mentioned RE-AIM dimensions, stakeholders were interviewed on hindering and fostering factors regarding reach, adoption, implementation as well as maintenance of Internet-based interventions for eating disorder patients and carers. The target groups of online interventions (patients and carers) were only asked about the factors for reach and implementation.

Potential facilitators were also asked in the online questionnaire to rate their perceived ratio between advantages and disadvantages on a scale of −5 (=much more disadvantages) to +5 (=much more benefits). Additionally, they were asked to rate their level of acceptance to integrate these interventions in the German health care system as well as their willingness to actively support the integration on a 10-point-scale (0 = not at all, 10 = absolutely).

The description of the development of the instrument of the overall stakeholder survey can be found in Nitsch *et al.*^36^ in this supplement.

### Recruitment and procedure

To examine the perspectives of potential users, eating disorder patients (P) and carers (C) of individuals suffering from an eating disorder were recruited to participate in focus groups via cooperating psychosomatic hospitals, psychotherapists in private practices, counselling centres and self-help group leaders. Furthermore, perspectives of potential decision-makers (DM) like politicians, health authority staff or health insurance staff were examined as they are important with regards to the implementation of online interventions. Representatives on the decision-making level were identified via personal contacts as well as official websites and subsequently invited by telephone inquiries to take part in a semi-structured interview. All focus groups and interviews were video or audio recorded. The focus groups and interviews were conducted after receiving informed consent for interview participation and recording from each participant. As a third relevant stakeholder group, facilitators respectively delivery staff (F) such as psychotherapists, psychiatrists or nurses were contacted by sending e-mails containing a study description as well as a link leading to an online survey to a mailing list of the Institute of Clinical Psychology and Psychotherapy of the TU Dresden. They were also asked to forward the link to other colleagues on the same field of activity.

### Data analysis

Quantitative online survey data were analyzed in IBM SPSS statistics 25.^38^ Focus groups and telephone interviews were transcribed verbatim in German. These transcripts as well as the answers of participating facilitators to open questions of the online-questionnaire were coded and analyzed using thematic analysis to identify superordinate categories of statements.^39^ In a first step, the transcripts were read from two coders. The subsequent steps included identifying related issues, creating initial codes, comparing results of both coders and re-coding if necessary. After that, final codes and themes were defined. Identified common themes were assigned to the following topics: experiences, attitudes, characteristics and influencing factors following the RE-AIM dimensions.

Ethical approval for the We Can trial was obtained on 01 December 2016 from the Institutional review board at the TU Dresden (Dresden, Germany), EK 500122016.

## Results

Five focus groups with a total of 20 patients (*N* = 3) and 10 carers (*N* = 2) were conducted in a psychosomatic clinic and two self-help counselling centres specialized to eating disorders. Each carer focus group consisted of five participants. Two patient focus groups consisted of seven participants, six participants could be included in the third patient focus group. The focus groups lasted between 48 and 74 minutes (mean: 56.20; SD: 10.77). Four decision makers agreed to take part and were interviewed on the phone with a duration between 21 and 42 min (mean: 29.50, SD: 9.33). Additionally, 41 potential facilitators filled out the online questionnaire.

All participants were recruited in Germany. A detailed description of the sample and all sociodemographic data collected is summarized in table 1.

**Table 1:** Sample description of all interviewed stakeholders (*N* = 75)

Participant characteristics—patient focus groups (*N* = 3)	
Gender	*N*
Total	20
Female	20
Male	0
Participant characteristics—carer focus groups (*N* = 2)	
Gender	*N*
Total	10
Female	9
Male	1
Kind of relationship	*N*
Parent	7
Sibling	3
Participant characteristics—online questionnaire (*N* = 41)	
Profession	*N*
Psychologist	18
Psychotherapist	8
Psychiatrist	2
Social worker	2
Nurse	2
Other	9
Years of experience	Mean (SD)
Years	9.40 (8.98)
Participant characteristics—telephone interviews (*N* = 4)	
Gender	*N*
Total	4
Female	2
Male	2
Stakeholder group	*N*
Health authority	2
Association of German Professional Psychologists (BDP)	1
Health insurance	1

Since the answers given by the different stakeholder groups show almost no differences, the results are presented not separately for each group. Statements by patients and carers will be presented as statements from the target group. If an aspect was mentioned by just one stakeholder group, it is specified in the text. To underline some statements, quotations will be included in the respective text passages.

### Experiences

About 71% (*N* = 29) of the participating facilitators affirmed in the online questionnaire that they have heard or read about online self-help interventions for eating disorder patients and carers before, but less than half (44%, *N* = 18) have looked into such a programme. Only a few (14.6%, *N* = 6) mentioned personal experiences, such as participating in an online self-help programme. The same number (14.6%, *N* = 6) of participants reported to have supported the implementation of such interventions.

None of the interviewed stakeholders on decision-making level as well as nobody from the carer focus groups stated previous experiences with Internet-based interventions. A few eating disorder patients had heard from Internet-based interventions before and only one of them reported previous participation in such a programme.

In general, the understanding of Internet-based self-help differs between participants. When asked for their experiences, some interviewed patients also mentioned WhatsApp groups, forums like ‘ProAna’ or websites providing experience-reports as used self-help services. One interviewed decision maker noted, that self-help cannot be provided via an Internet-based program, since ‘self-help is based on self-organization of people affected’.

### Attitudes

To examine their attitudes, participants were asked to state advantages and disadvantages with regard to online interventions for eating disorder patients and carers. Overall, we identified five main advantages and four main disadvantages.

#### Advantages

1. Anonymity: anonymity was reported as a major advantage in all stakeholder groups, e.g. to overcome barriers like fear of stigmatization.
  When looking back upon the beginning of the disease, while being on a waiting-list for eight weeks, it would have been helpful to have a safe space for chatting anonymously with other patients. (P)
2. Bridging waiting time: the advantage to bridge waiting time before getting access to outpatient or inpatient treatment was stated from decision makers and facilitators as well as in the patient focus groups.
3. Availability: all included stakeholder groups considered flexibility in terms of location and time as an advantage and therefore Internet-based interventions as a low-threshold service. One carer considered Internet-based interventions as ‘help whenever it will be needed’. It was also mentioned that online programmes can be used flexible as it is not necessary to make an appointment. Some participants from the target group and the decision-making level also emphasized the reach of underserved populations, e.g. in rural areas. Furthermore, an Internet-based programme was considered to provide help and information timely.
4. Cost-effectiveness: the participating facilitators stated the cost-effectiveness of Internet-based programmes as a large benefit compared with costs that arise through face-to-face services.
5. Use of new technologies: to provide internet-based interventions, modern technology can be used that is widely available and frequently used by the majority of the people. It offers various possibilities like interactivity. Stakeholders from the target group perceived it as an advantage to write about problems instead of talking about problems.

#### Disadvantages

1. Limitations by symptom severity: stakeholders on decision-making level noted that Internet-based interventions are only applicable to individuals with a certain degree of symptom severity. Specifically, patients expressed fears of receiving insufficient support. Furthermore, stakeholders stated it would be impossible to react in emergency situations.
  It is a disadvantage that it can only be used to a limited degree of the disease severity. For example, when the BMI is below a specific limit, direct medical intervention is necessary […]. (DM)
2. Lack of personal contact: due to the lack of personal contact as well as the related lack of verbal and non-verbal communication, experiencing emotions of one’s counterpart can only occur to a limited degree. Carers expressed the fear of feeling alone or isolated. All stakeholders stated the fear of difficulties with regard to developing a therapeutic relationship, monitoring of intended programme use, success monitoring, difficulties to assess the course of disease as well as misunderstandings due to the missing face-to-face contact. Also, faking can be an issue in Internet-based programmes, as the target group assessed it as almost impossible to check the seriousness of his counterpart. As a further disadvantage, the lack of customizability was mentioned. With regard to disadvantages, anonymity is not only seen as a benefit and can lead to a constraint of commitment. Internet-based interventions seem to have a non-obligatory character, possibly leading to a lack of motivation as well as high drop-out rates.
  Anonymity can also be a disadvantage. It is non-obligatory and I can state whatever I want. The programme wouldn’t notice it. And it can’t have the same effect as a face-to-face meeting with a therapist who will notice my feelings […]. (DM)
3. Availability: the low-threshold availability of using the programme anytime and at every place was also seen as a possible disadvantage. The potential facilitators mentioned concerns regarding postponing or rejecting of visiting a physician or therapist. The target group additionally stated the use of mobile phone/computer as a stressor because it is difficult to switch it off unlike leaving after a face-to-face treatment unit.
4. Data protection: data safety concerns were mentioned by many stakeholders. Especially potential users of such interventions expressed fears of data misuse.

Overall, the participating facilitators stated in the online questionnaire that Internet-based interventions for eating disorder patients and their carers have more benefits than disadvantages (mean = 2.56; SD = 1.45). Further, they rated their level of acceptance to integrate these interventions in the German health care system as high (mean = 8.41; SD = 1.80) and their willingness to actively support the integration as medium to high (mean = 6.72; SD = 3.03).

### Underserved groups of persons

All interviewed stakeholders considered Internet-based interventions especially useful for younger people. Stakeholders stated people living in underserved and rural areas might benefit from Internet-based interventions. Additionally, stakeholders on decision-making level considered these interventions to be useful to bridge waiting-time or to provide aftercare support. According to the target group, people fearing face-to-face interventions might benefit from online programmes. They also mentioned to provide these interventions in consideration of disease severity and therefore only to people being in a stable phase.

### Characteristics

All participants were asked about the design and necessary features of an Internet-based intervention for eating disorder patients and carers. Five main characteristics were identified:

1. Tools: an online programme should consist of various kinds of interactive tools like practical exercises, a (group) chat function to enable an exchange with other carers, patients and professionals as well as motivational elements like personal feedback. Decision-makers and facilitators stated using adherence messages as a further motivational tool, e.g. offered through an app-based reminder function. However, the integration of reminder messages was discussed controversially by the target group.
  I like these reminders. Well, to receive this e-mails […] reminding me to do something. (P)
  To be honest, I always perceived them as annoying. It would be good to have the opportunity to turn them off. (P)
2. Design: the design of an Internet-based programme should be attractive, reliable and user-friendly, i.e. comprehensible, short and precise. Additionally, all stakeholders emphasized the importance of a responsive design when using the mobile version. Furthermore, patients would prefer a hidden programme logo to prevent others to draw any conclusions regarding their disease.
3. Themes: the target group and stakeholders on decision-making level mentioned the aspect of adaptability, e.g. on the individual level of the users’ knowledge, as well as the option to choose personal routes. Topics that should be included are psychoeducation in general and disease-specific, how to cope with stress, promotion of resources and self-care. The target group also expressed a desire for case examples as well as information on available services and self-help groups.
4. Contact options: providing contact to a professional who gives feedback on open questions or on the individual progress in the online intervention is considered as necessary in terms of adherence. Personal contact should take place in the form of scheduled appointments, e.g. via e-mail or a live chat-function. In general, a contact person should be available at scheduled times and in emergency situations via a helpline or support via e-mail.
5. General: a few participating facilitators mentioned the importance of economic factors like costs or expenditure of time. The target group expressed the desire to use the Internet-based intervention at no or low cost. The intervention should also be evidence-based and, as mentioned by the target group, even be available after the acute phase of the disease. Most of the stakeholders of the target group and of the facilitators stated it as essential to ensure an anonymous participation as well as data safety and to ensure the option to delete participant data if requested.
6. Influencing factors following the RE-AIM dimensions
  - Reach: this dimension refers to the question how the targeted population can be reached. To announce an intervention, facilitators and decision-makers stated fostering factors like promotion of the programme, e.g. through health care professionals, health ministry, health insurances, counselling centres, universities or public authorities to build confidence. Continuous dissemination activities were considered to have to be done to increase reach. To get people using the programme, especially factors with regard to usability and low-threshold access, salience compared with other programmes, reliability (e.g. through quality criteria), transparency regarding the provider, financing by health insurances, providing contact to professionals as well as other patients/carers within the programme and responsive design were discussed as fostering factors. Regarding hindering factors, stakeholders of the target group stated the obligatory use, missing data safety, the missing opportunity to delete the account or poor quality of provided information.
    Such a specific programme has to be promoted purposefully […] because everyone can be present on the internet […] and there can also be dubious providers what can cause harm […]. (DM)
  - To get people using the programme, especially factors with regard to usability and low-threshold access, salience compared with other programmes, reliability (e.g. through quality criteria), transparency regarding the provider, financing by health insurances, providing contact to professionals as well as other patients/carers within the programme and responsive design were discussed as fostering factors. Regarding hindering factors, stakeholders of the target group stated the obligatory use, missing data safety, the missing opportunity to delete the account or poor quality of provided information.
    Such a specific programme has to be promoted purposefully […] because everyone can be present on the internet […] and there can also be dubious providers what can cause harm […]. (DM)
  - Adoption: Adoption refers to the number of organizations who are willing to deliver the programme. Participating facilitators emphasized the following factors as fostering: usefulness, integrability in the daily workflow and usability for participants and delivery staff. All participating stakeholders stated that the programme should also be evidence-based and certified and has to provide financial and practical benefits in terms of facilitation of the workload for delivering people. Furthermore, the involvement of health insurances, health care professionals and services and the health ministry would have to be considered, as they were seen as potential distributors and supporters. With regards to hindering factors, potential facilitators mentioned the experience of Internet-based interventions to be in competition with face-to-face care and a conflict of interest (not further specified) regarding the intervention.
    […] it must fit into the daily routine of the professionals who shall use the programme. This will lead to an increased willingness to use the programme and to work with it. (DM)
  - Implementation: to implement an Internet-based programme as intended was seen as essential to include the programme into the daily workflow of the delivery staff. Stakeholders also stated that the provider has to secure financing and to offer manuals and personal support for the delivery staff. The programme should be conducted by trained and specialized staff.
    I will emphasise the aspect of practical relevance. It’s not useful when it is not compatible with the daily workflow of physicians and therapists […] and it would be very helpful to have a manual or a guideline or something like that to ensure implementation as planned […]. (DM)
  - Maintenance: maintenance refers to the aspect of sustainability and therefore to the extent to which the Internet-based programme becomes part of the organizational routine. It also refers to the long-term effect of the programme on outcomes of the individuals. With regard to foster maintenance, two main factors were discussed. The first factor, quality management, includes aspects like continuous programme evaluation and adaptation, cost-benefit analyses and a periodic training of delivery staff. To include user feedback in the programme evaluation and adaptation process was seen as indispensable. The second factor refers to feasibility and contains secured financing and the implementation of the intervention as a standard procedure. As hindering factors, stakeholders stated especially no recognizable benefit and a lack of support by health insurance companies.
    The evidence of efficacy will play a significant role. […] And I think it is important that from the beginning the focus is on a continuous development […]. (DM)

## Discussion

### Principal Findings

The aim of this study was to identify stakeholders’ experiences with and attitudes towards Internet-based interventions for eating disorder patients and carers of people suffering from an eating disorder in Germany. A similar study was conducted in the UK, the corresponding article is also published in this special issue.^40^

Most stakeholders of the target groups as well as on decision-making level mentioned to have no or little experiences with the above mentioned online programmes. In contrast, a lot of participating potential facilitators had at least heard or read about online interventions. Therefore, it has to be considered that most answers were given primarily from a theoretical point of view. Furthermore, it must be kept in mind that on all stakeholder levels, we found different opinions of what an Internet-based intervention is like. This is mainly due to the heterogeneity of the several stakeholder groups.

Consistent with previous findings,^12^^,^^28^ stakeholders noted advantages like the use of modern technology, accessibility at any place and time and therefore the ability to reach underserved populations, e.g. in rural areas. Since carers of eating disorder patients are often under time pressure and therefore do not have the chance to seek and use regular face-to-face help, an Internet-based intervention offers the opportunity to achieve help whenever and wherever needed. This also applies to eating disorder patients who cannot seek face-to-face treatment due to other obligations during the week.

Furthermore, stakeholders underlined the cost-effectiveness as well as the availability compared with face-to-face interventions. Specifically, the long waiting periods for face-to-face treatment were discussed by almost all stakeholders. It is well-known that a large number of eating disorder patients are waiting several weeks or even several months for face-to-face treatment.^2^ Stakeholders mentioned that Interned-based interventions can help to bridge waiting time via offering support during this period. Dölemeyer *et al.*^13^ emphasized anonymity as a benefit of online programmes. This was also confirmed in our stakeholder survey. All groups mentioned anonymity as a major benefit of Internet-based interventions, e.g. as it can facilitate seeking and utilizing help while overcoming barriers like social anxiety.^27^ When it comes to disadvantages, anonymity was also mentioned as an adverse factor. An Internet-based intervention seems to be non-obligatory which can result in a decreased motivation as well as an increase of drop-out rates. Additionally, stakeholders expressed concerns that the lack of personal contact can also impair the development of a therapeutic relationship. Further concerns were expressed regarding data safety, limitations by disease severity and an almost unrestricted availability which may hinder patients to seek face-to-face treatment. The last point is consistent with previous findings, that psychologists reported fear of conventional face-to-face treatment being replaced by online treatments and were therefore afraid of losing their work.^12^ Andersson and Titov,^12^ therefore, stated that online-based interventions should rather be considered as a complement to existing services. To reduce concerns and negative attitudes towards Internet-based interventions, more educational work should be done by providing more detailed information about using new technologies when offering psychological treatment.

Stakeholders on all levels stated several characteristics that Internet-based interventions should have: tools like a chat function to enable contact with other participants or professionals. For example, personal feedback and support were seen as indispensable to increase motivation and adherence of the users. These needs were also previously reported.^27^ To overcome concerns regarding data safety, providing information on data protection as well as the option to delete user data whenever wanted are perceived as necessary. Further information should be given on the provider of the programme and its evidence base. Users would appreciate it to see a kind of certificate of approval. Otherwise, potential users will experience fear of choosing an untrustworthy programme and of receiving inadequate support. Beside these characteristics, the programme should be attractive and user-friendly as well as suitable for mobile devices.

With regard to the first RE-AIM dimension, reach, stakeholders perceived it as positive to get to know the programme via prominent services and authorities like health care insurances or universities as this would increase trust in the programme. This might help overcome barriers in terms of concerns about seriousness and reliability. Therefore, it is very important to address prejudices of disseminating and delivering stakeholders by providing information on Internet-based interventions in order to reduce these prejudices. Additionally, to promote trust in the intervention, data safety has to be guaranteed. Another issue mentioned as important is the need for customizability. Potential users would prefer tailored interventions to take individual circumstances into account. Beside these aspects, stakeholders stated that they would not use the online programme if it was not offered for free. Therefore, stakeholders suggested financing the use of Internet-based programmes by health insurances.

When it comes to factors that foster adoption of such programmes, stakeholders especially emphasized the need for reducing or at least not increasing the workload of the delivery staff (e.g. psychologists). They were willing to deliver a programme if it is evidence-based, effective, if it fits into the daily workflow and provides practical benefits. Considering Internet-based interventions as a replacement for face-to-face interventions was stated as an important factor that reduces the willingness to deliver these programmes. As mentioned above, these concerns can be addressed by education and more systematic training.^30^ It can be seen as a major challenge to create a broad awareness that Internet-based interventions can help to overcome healthcare gaps and provide additional benefits as mentioned above. However, in the present study potential facilitators assessed Internet-based interventions to have more assets than drawbacks. Most of them would also endorse the integration as well as actively support the implementation of these interventions into the German health care system. These positive attitudes should be considered as a benefit when it comes to promote adoption.

To implement an online intervention as intended, stakeholders regarded the training of delivery staff as crucial. Additionally, personal support by the provider as well as a manual should be provided. These suggestions were in accordance with findings reported by Wells *et al.*^28^

Considering the last RE-AIM dimension, maintenance, stakeholders emphasized the importance of including Internet-based interventions into routine care. It is therefore necessary to secure financing and to continuously evaluate and adapt the interventions. Providers should also bear in mind to regularly assess and incorporate user feedback.

### Limitations and future directions

As mentioned above, most stakeholders of the target group reported to have no previous experiences with Internet-based interventions. Therefore, it has to be taken into account that their answers were given from a more theoretical perspective. This also applies to stakeholders on the decision-making level. Partly, they stated to have no idea how an online programme could look like, thus being possibly more reserved. When conducting a stakeholder survey in the future, a definition of Internet-based self-help needs to be given to the participants to avoid large discrepancies regarding their knowledge. The participating potential facilitators mentioned low experience with the use of online interventions which restricts the generalizability with regard to trained delivery staff. Experts in the field of Internet-based interventions might have a different opinion. Since experiences and perspectives of health care providers are influenced by factors such as kind of institution, therapy modality or clinical experience, these factors also have to be assessed in future studies. According to this, a more detailed description (e.g. age, eating disorder diagnosis) of the target sample should be done.

Furthermore, since we were not able to assess attitudes of non-responders the relatively small sample may consist of participants with specific attitudes and thus be biased.

Future research should focus on country-specific differences regarding the integration of Internet-based interventions into routine care, i.e. on questions like how to include it into daily routine of health care professionals, how to improve dissemination and how to secure financing, since the health care systems as well as the current incorporation of E-mental health vary between countries. Additionally, characteristics of Internet-based interventions proposed by stakeholders need to be implemented and evaluated.

With regard to the prevailing COVID-19 pandemic, further surveys have to be conducted to examine changes in attitudes, experiences and expectations of different stakeholder groups. It is likely that both users and providers of online mental health care reported additional advantages, but also disadvantages and changed their perspective towards Internet-based interventions and, as a result, also their use of those services in the future.

## Conclusion

To the best of our knowledge, this is the first study to focus on the view of three stakeholder levels: First, stakeholders at the governing level were interviewed since they are important decision-makers. Second, health care professionals were assessed because they are representatives of the delivery staff and therefore potential distributors and facilitators. As a third group, stakeholders of the target group were assessed as they represent potential users (patients and carers). Furthermore, this study is the first to examine the perspectives of carers of individuals suffering from an eating disorder in Germany on online self-help interventions.

Most stakeholders reported to have no or little experiences with Internet-based interventions for eating disorder patients and their carers. Asked for their attitudes, they reported a large number of advantages, e.g. low-threshold, anonymity and flexibility in Internet-based interventions. Overall, stakeholders considered online programmes to have more advantages than disadvantages. The stated disadvantages and prejudices can be addressed through education in terms of providing information on such interventions. Efforts should therefore be put in building awareness for the many benefits like cost-effectiveness and support during waiting periods for outpatient or inpatient treatment. When offering an online programme, several issues have to be considered. First, building trust by providing the programme via renowned institutions as well as providing a cachet will enhance peoples’ willingness to use the programme. Furthermore, the individuality of potential users with regard to their personal needs and circumstances has to be taken into account. Thus, future Internet-based interventions should rather be tailored individually and consist of a modular system. Second, besides education about online interventions in general, specific workshops should be offered to train professionals in order to further reduce uncertainties and prejudices. To increase professionals’ willingness to deliver Internet-based interventions, it is also necessary to make the interventions useful and effort-saving. Finally, when developing and implementing online interventions, the aspect of sustainability has to be considered to embed the programme into routine care and to generate positive long-term effects in users.

## Funding

This project has received funding from the European Union’s Horizon 2020 research and innovation programme under grant agreement No. 634757.

**Key points**

- Stakeholders are aware of Internet-based self-help interventions in general, but have no or little personal experiences.
- Anonymity, flexibility, low-threshold access, cost-effectiveness and the opportunity to bridge waiting-time were seen as major advantages of Internet-based self-help interventions.
- Stakeholders stated the need for interventions which are evidence-based, user-friendly, tailored and provide personal support.
